# SR‐717, a Non‐Nucleotide STING Agonist, Displayed Anti‐Radiation Activity in a IL‐6 Dependent Manner

**DOI:** 10.1096/fj.202403127R

**Published:** 2025-05-31

**Authors:** Duo Fang, Wanli Duan, Xuanlu Zhai, Liao Zhang, Jiayan Fang, Keer Jiang, Jianpeng Zhao, Yue Fu, Lan Fang, Lu Pei, Cong Liu, Jicong Du, Jianming Cai, Fu Gao

**Affiliations:** ^1^ Department of Radiation Medicine, Faculty of Naval Medicine Naval Medical University Shanghai China; ^2^ School of Public Health and Management Wenzhou Medical University Wenzhou China

**Keywords:** IL‐6, intestinal stem cells (ISCs), IR‐induced intestinal injury, STING, TLR2

## Abstract

Ionizing radiation (IR) induced damages are common complications of radiotherapy for tumors, severely limiting the intensity and therapeutic efficacy of the radiotherapy program. Emerging data indicated that the cGAS‐STING pathway has paradoxical effects on IR‐induced damage. SR‐717, as a non‐nucleotide, small‐molecule stimulator of interferon genes (STING) agonist, has been proven that it could activate the STING signaling pathway. In this work, we try to explore the radioprotection of the STING signaling pathway and figure out whether SR‐717 could be a potential intestinal radioprotective agent. C57BL/6 mice were intraperitoneally treated with SR‐717 or normal saline (NS). By analyzing the survival rate, body weight, and the number of peripheral blood cells after IR exposure, we found that SR‐717 improved the survival rate and body weight of mice, protected the intestine from IR‐induced damage as well as hematopoietic damage, and promoted the regeneration of intestinal stem cells (ISCs). Cell viability and apoptosis after irradiation were detected after stimulation of MODE‐K cells with SR‐717 or PBS. We found that SR‐717 increased cell viability and inhibited apoptosis in vitro. The mechanism of SR‐717 in intestinal radiation protection was investigated by RNA‐seq. The results of RNA‐seq and qRT‐PCR suggested that SR‐717 significantly activated the immune system via the STING‐IL‐6 signaling pathway. In addition, we discussed the role of TLR2 in SR‐717‐mediated anti‐radiation activity, and TLR2 deletion significantly reversed the radioprotective effects of SR717. In conclusion, we proved STING signaling activation displayed anti‐radiation activity and found SR‐717 displayed anti‐radiation activity via the STING‐IL‐6 signaling pathway, suggesting SR‐717 could be a potential intestinal radioprotective agent.

## Graphical abstract

SR‐717 displayed anti‐radiation activity in a IL‐6 dependent manner.

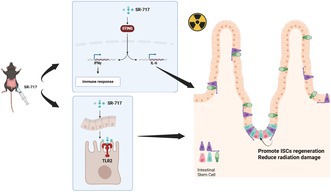

## 1 Introduction

The intestine is the largest organ to absorb the nutrients and water in the human body, which not only has the functions of digestion and absorption, but also provides effective protection against the invasion of harmful substances and pathogens in the environment [1]. Intestinal epithelial cells are constantly self‐renewing by intestinal stem cells (ISCs). ISCs are located at the base of the crypt and produce epithelial cells which are renewed about every 5 days [2, 3]. The intestinal epithelium is extremely sensitive to IR exposure [4, 5]. Currently, there is a lack of an effective method for IR‐induced intestinal injury. Herein, we attempted to find novel and potential intestinal radioprotective agents and explore the mechanism behind ISCs regeneration after IR exposure.

The cGAS‐STING pathway can recognize the abnormal presence of double‐stranded DNA (dsDNA) in the cytoplasm and is a crucial part of the innate immune response. cGAS‐STING pathways can rapidly generate an immune response to invasive pathogens and their own necrosis, apoptosis, and damaged tissue, building the first defensive line to protect the body [6, 7, 8]. Emerging data indicated that the cGAS‐STING pathway has paradoxical effects on IR‐induced damage. Leibowitz et al. showed that STING loss had no effect on IR‐induced DNA damage or intestinal crypt apoptosis but inhibited IFN‐β production, local inflammation, and subsequent crypt regeneration. They also found that cGAS deletion or IFNAR1 deletion inhibited IR‐induced intestinal crypt inflammation and regeneration. Moreover, impaired intestinal regeneration and survival in STING‐deficient mice are fully rescued by a single IFN‐β treatment [9]. Hayman et al. revealed that STING loss confers resistance to IR‐induced damage [10]. Du et al. indicated that cGAS‐STING deficiency could attenuate radiation‐induced hepatic steatosis and inflammation in mice, and blockade of type 1 IFN signaling protected mice from radiation‐induced liver injury [11].

SR‐717, a non‐nucleoside STING agonist, has been proven that it could significantly inhibit tumor growth, prevent metastasis, induce the presentation of tumor molecules to the immune system, and strongly increase the levels of CD8^+^ T cells and NK cells around the tumor [12]. In our work, we found that the activation of STING could protect IR‐induced damage. Moreover, we found SR‐717 protected the intestine against IR‐induced damage via the STING‐IL‐6 signaling pathway in vivo and in vitro, which suggested that SR‐717 may be a potential intestinal radioprotective agent.

## 2 Materials and Methods

### 2.1 Chemicals and Reagents

The detailed materials were provided in the Supporting Information S1.

### 2.2 Animals and Treatment

C57BL/6 mice were obtained from China Academy of Science (Shanghai, China). Wild‐type (WT) littermates and TLR2 KO mice, 6–8 weeks old, were purchased from Cyagen (Jiangsu, China). Mice were kept under standard conditions in a laboratory animal room. The experiments were approved by the Laboratory Animal Center of the Naval Medical University, China, in conformance with the National Institute of Health Guide for the Care and Use of Laboratory Animals. The mice were treated with SR‐717 (30 mg/Kg) via peritoneal injection 18 h and 2 h before IR.

C176 was used to inhibit STING in mice. The mice were treated with C176 (MedChemExpress, HY‐112906, 5 mg/kg) via peritoneal injection 18 h and 2 h before IR.

Anti‐mouse IL6‐InVivo (Selleck, A2118, 5 mg/kg) was used to inhibit IL‐6 signaling. Anti‐mouse IL6‐InVivo was administered as an intraperitoneal injection 2 h before IR and on Days 1–4 after IR.

### 2.3 Cell Culture and Treatment

MODE‐K were purchased from American Type Culture Collection. MODE‐K was cultured in RPMI 1640 medium containing 10% FBS at 37°C in a 5% CO_2_ humidified incubator. The cells received 20 μM SR‐717 before IR.

### 2.4 Irradiation

^60^Co (Naval Medical University, China) was used for all experiments. The mice were irradiated at 8.0 and 9.5 Gy to establish the Total Body Irradiation (TBI) model. Irradiation of MODE‐K cells and the intestinal organoid was performed with the designated dose.

### 2.5 Histological Examination

The intestine was removed and collected from mice and then fixed in 4% paraformaldehyde. Hematoxylin and Eosin (HE) were performed according to the manufacturer's instructions.

### 2.6 Intestine Immunofluorescence

The Expression of OLFM4 and LYZ1 in the mice intestine was detected by using immunofluorescence analysis. The intestine was removed and fixed in 4% paraformaldehyde for 25 min and permeabilized in 0.5% Triton X‐100 for 10 min. After blocking in BSA, the intestinal tissues were stained with antibodies, followed by the secondary antibody (1:1000). The images were obtained with a fluorescent microscope. The mean fluorescence intensity (MFI) of OLFM4 and LYZ1 was measured by Image J (National Institutes of Health, Bethesda, MD, USA).

### 2.7 Intestinal Organoid Culture

The intestine was removed from the mice, cut longitudinally, and rinsed with cold PBS. The villi were gently scraped off, and the remaining tissue was washed about 20 times with cold PBS containing 1% penicillin–streptomycin. The tissue was cut into 2–3 mm pieces, transferred to 15 mM EDTA/PBS, and incubated for 2 h at 4°C. After incubation, the tissue fragments were shaken vigorously and centrifuged three times at 300 rpm/min with cold PBS for 5 min each time. The isolated crypts were then embedded in Matrigel and cultured in crypt culture medium. After half an hour, 500 μL organoid growth medium was dispensed to each well. Organoids were irradiated 24 h after inoculation. On Day 7 after IR, mature organoids were observed under a microscope. For radiation response assays, the surface area and budding condition of intestinal organoids were measured by using Image J.

### 2.8 RNA Sequencing and Functional Enrichment Analysis

Total RNA was isolated from the intestine of mice using Trizol (Invitrogen, USA) 24 h after radiation. NanoVue (GE, USA) was used to assess RNA purity. Sequencing was performed at Oebiotech (Shanghai, China) with the Illumina HiSeq 2500 system. Prior to sequencing, the raw data were filtered to produce high‐quality clean data. All the subsequent analyses were performed using clean data. All the differentially expressed genes (DEGs) were used for the analysis of heat maps and KEGG ontology enrichment analyses.

### 2.9 Western Blot

Total protein from the MODE‐K cells was extracted using mammalian protein extraction reagent. Cells were treated as indicated and harvested for protein extraction, and then analyzed by western blotting to detect β‐Actin (Cell Signaling Technology, 4970S, 1:1000), STING (Cell Signaling Technology, 13647, 1:1000), p‐STING (Cell Signaling Technology, 72971, 1:1000), p‐TBK1 (Cell Signaling Technology, 5483, 1:1000), p‐p65 (Cell Signaling Technology, 8242T, 1:1000), STAT3 (Proteintech, 10253‐2‐AP, 1:2000), p‐STAT3 (Proteintech, 28945‐1‐AP, 1:1000), TLR2 (Cell Signaling Technology, 13744, 1:1000) and Myd88 (Cell Signaling Technology, 4283, 1:1000). The secondary antibody was purchased from Servicebio (GB23301, GB23303, 1:5000).

### 2.10 Quantitative Real‐Time PCR (qRT‐PCR)

RNA was extracted using Trizol (Invitrogen, USA) according to the manufacturer's instructions. RNA quality and quantity were determined using Nanodrop ND‐1000 Spectrophotometer (Thermo Fisher Scientific, USA). Each RNA sample had an A260:A280 ratio > 1.8 and an A260:A230 ratio > 2.0. Reverse transcription assay was performed using the Hifair 1st Strand cDNA Synthesis SuperMix (11202ES08, Yeasen). The cDNA was then analyzed by qRT‐PCR using Hieff qPCR SYBR Green Master Mix (11202ES08, Yeasen). The list of primers is shown in Supporting Information S2.

### 2.11 Statistical Analysis

Each experiment was independently repeated three times and the representative data are shown. All the values were expressed as mean ± the standard errors of means. Two‐tailed Student's *t*‐test was used to analyze the differences between two groups. One‐way ANOVA was employed to analyze the differences among three groups. Kaplan–Meier analysis was applied to estimate the difference in overall survival between two groups. The data were analyzed using SPSS ver. 19 software (IBM Corp, Armonk, New York, USA). *p* < 0.05 was considered statistically significant.

## 3 Results

### 3.1 The Activation of STING has a Significant Radioprotection on IR‐Induced Damage

**FIGURE 1:**
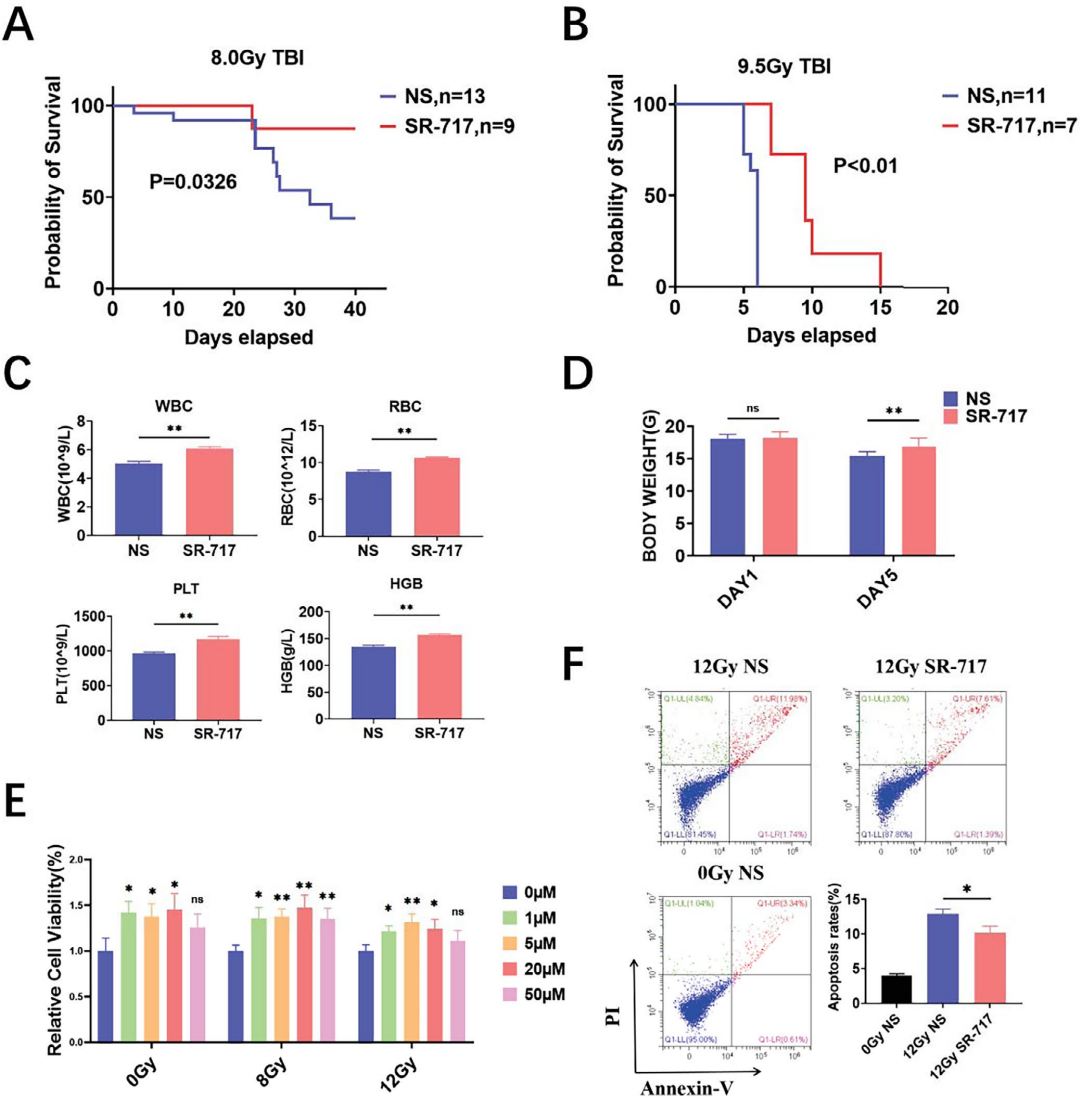
The activation of STING has a significant radioprotective effect on IR‐induced damage. (A) The survival of mice after 8.0 Gy TBI. (B) The survival of mice after 9.5 Gy TBI. (C) Changes of WBC, RBC, PLT, and HGB in the blood of mice after 9.5 Gy TBI (*n* = 3). (D) Weight changes of mice after 9.5 Gy TBI (*n* = 3). (E) The radioprotective effect of SR‐717 on MODE‐K cell viability was measured by CCK‐8 (*n* = 6). (F) The radioprotective effect of SR‐717 on MODE‐K cell apoptosis was detected by flow cytometry (*n* = 3). Data are represented as mean ± SEM. *p < 0.05, **p < 0.01.

Stimulator of interferon genes (STING) links innate immunity to biological processes ranging from antitumor immunity to intestinal homeostasis. SR‐717, a non‐nucleotide, small‐molecule STING agonist, was used to trigger STING signaling. Firstly, C57BL/6 mice were intraperitoneally treated with SR‐717 or NS at 18 and 2 h before 8.0 Gy or 9.5 Gy total body irradiation (TBI). Then the survival rate and weight changes were assayed. We found SR‐717 prolonged the average survival time of irradiated mice under TBI (Figure 1A,B). SR‐717 also played a protective effect on the body weight change (Figure 1D). The hematopoietic system is sensitive to IR exposure. Blood routine results showed that SR‐717 increased the number of WBC, RBC, HGB, and PLT (Figure 1C). In addition, MODE‐K cells were used to detect cell viability and apoptosis at 24 h after irradiation. The results suggested that SR‐717 could significantly increase cell viability and decrease the apoptosis rate after IR (Figure 1E,F). Taken together, these results provided that SR7‐7 displayed anti‐radiation activity in vivo and in vitro, which suggested the activation of STING has a significant radioprotective effect on IR‐induced damage.

### 3.2 SR‐717 Alleviated IR‐Induced Intestinal Damage in Mice

**FIGURE 2:**
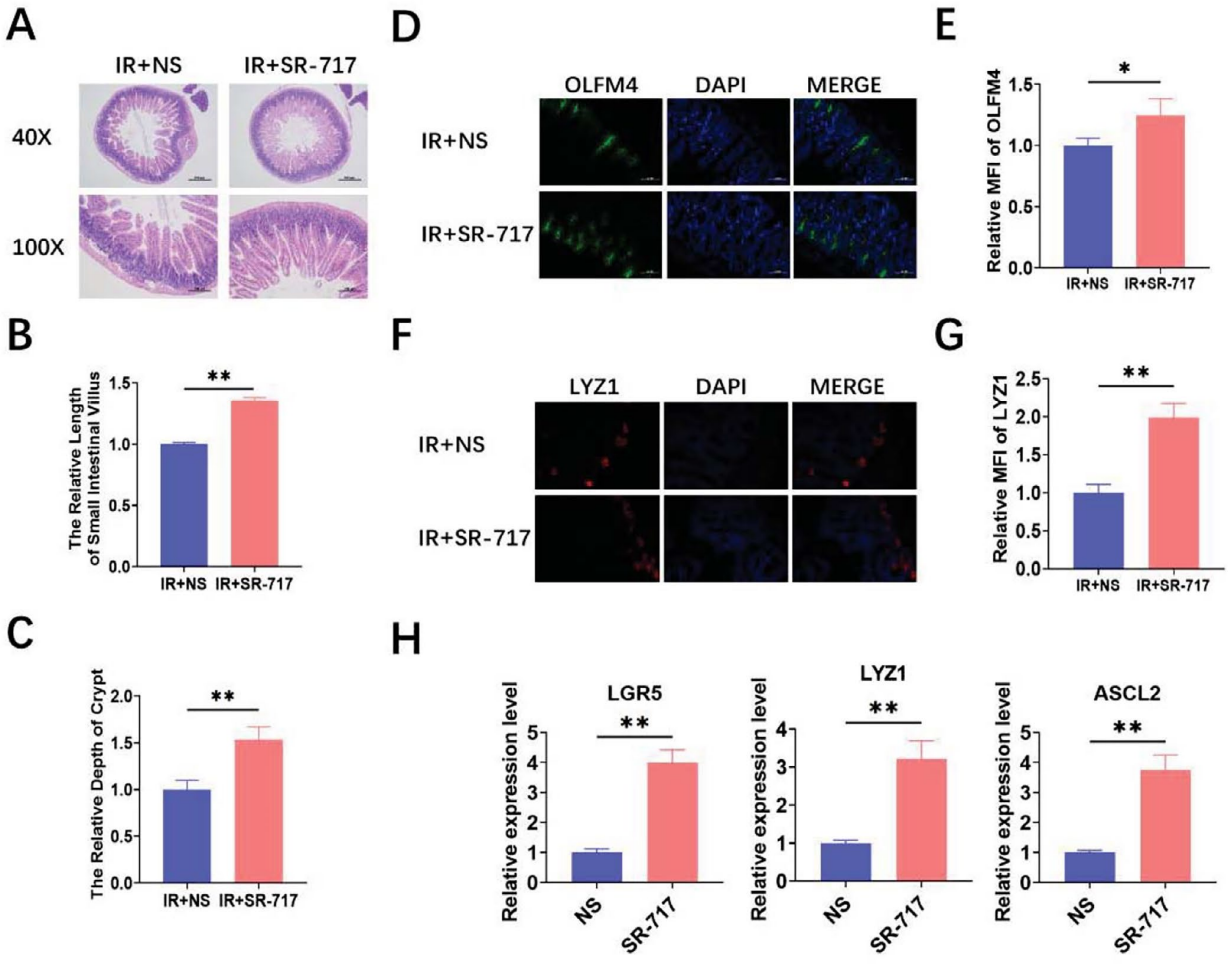
SR‐717 alleviated IR‐induced intestinal damage in mice. Mice were intraperitoneally treated with SR‐717 or NS before 9.5 Gy TBI. (A) Representative images of HE staining intestinal sections at Day 3.5 after 9.5 Gy TBI (*n* = 3). (B) The relative villus length (*n* = 3). (C) The relative crypt depth (*n* = 3). (D) Representative images of OLFM4 immunofluorescences at Day 3.5 after 9.5 Gy TBI (*n* = 3). (E) The relative fluorescence intensity of OLFM4 per section (*n* = 3). (F) Representative images of LYZ1 immunofluorescences at Day 3.5 after 9.5 Gy TBI (*n* = 3). (G) The relative fluorescence intensity of LYZ1 per section (*n* = 3). (H) RNA obtained from mice intestines was used to perform the qRT‐PCR of LGR5, LYZ1, and ASCL2 (*n* = 3). Data are represented as mean ± SEM. *p < 0.05, **p < 0.01.

The intestine is sensitive to IR exposure. Next, the intestinal radioprotection of SR‐717 was assayed. The intestines of mice were extracted at 3.5 days after 9.5 Gy TBI for pathological analysis. As a result, the intestinal damage degree of mice in the SR‐717 group was reduced significantly compared with the NS group (Figure 2A). The crypt depth and villus length were both increased compared with those in the NS group (Figure 2B,C). ISCs are susceptible to pathogenic environmental factors such as chemical factors, pathogens, and ionizing radiation. ISCs can rapidly respond to stressful insults and this response can be found after IR exposure. Paneth cells have been verified to have a significant niche effect in supporting ISCs through intercellular contact and the secretion of factors such as epidermal growth factor (EGF), Dll4, and Wnt3a. In addition, paneth cells, as a reserve ISCs population, can restore Lgr5^+^ ISCs through dedifferentiation during acute injury, thus promoting tissue regeneration [13]. Therefore, we evaluated the ISCs marker OLFM4 [14] and Paneth marker LYZ1 [15] in the mice intestine. We found the levels of OLFM4 (Figure 2D,E) and LYZ1 (Figure 2F,G) were increased in the SR‐717 group after 9.5Gy TBI. By extracting intestinal RNA from irradiated mice, we found that the expression levels of ISCs markers, including LGR5, LYZ1, and ASCL2, were remarkably increased in the SR‐717 group (Figure 2H). In sum, these results suggested that SR‐717 alleviated IR‐induced intestinal damage in vivo.

### 3.3 SR‐717 Promoted ISCs Regeneration After IR

**FIGURE 3:**
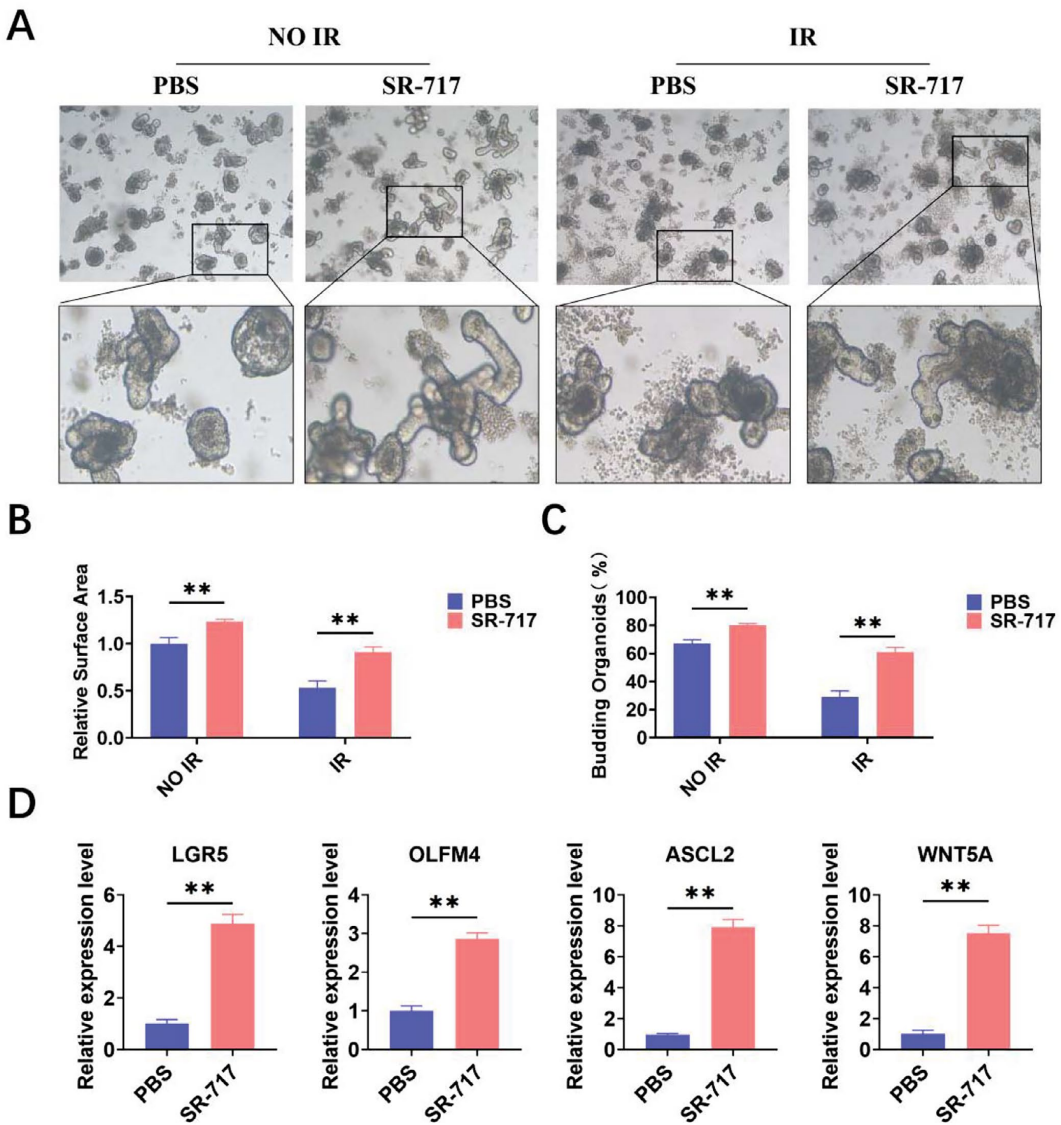
SR‐717 promoted ISC regeneration after IR. (A) Intestinal crypts were isolated from WT for organoid formation assay. (B) The relative surface area was analyzed and measured by using Image J. (C) The budding rate of intestinal organoids was analyzed and measured by using Image J. (D) RNA obtained from the intestinal organoids after IR was used to perform the qRT‐PCR of LGR5, OLFM4, ASCL2, and WNT5A(*n* = 3). Data are represented as mean ± SEM. **p < 0.01.

Intestinal organoids can simulate the complex structure and function of the intestine, and the proliferation of intestinal stem cells can be studied in vitro [16, 17]. Therefore, we used intestinal organoids to investigate the effect of SR‐717 on ISCs. Intestinal crypts were extracted from mice for intestinal organoid culture. Intestinal organoids were stimulated by SR‐717 or PBS 12h before 6 Gy IR exposure. The intestinal organoid results suggested that SR‐717 promotes the regeneration of intestinal organoids (Figure 3A). The relative area and budding rate of the SR‐717 group were improved compared to the control group (Figure 3B,C). Subsequently, we extracted intestinal organoid RNA after IR for verification and found that SR‐717 increased the expression of ISCs in intestinal organoids. In addition, SR‐717 also increased the key regulatory elements in the Wnt signaling pathway (Figure 3D). In sum, these results showcase that SR‐717 can promote ISC regeneration in vitro.

### 3.4 SR‐717 Activates the Immune System via STING‐IFN Signaling

**FIGURE 4:**
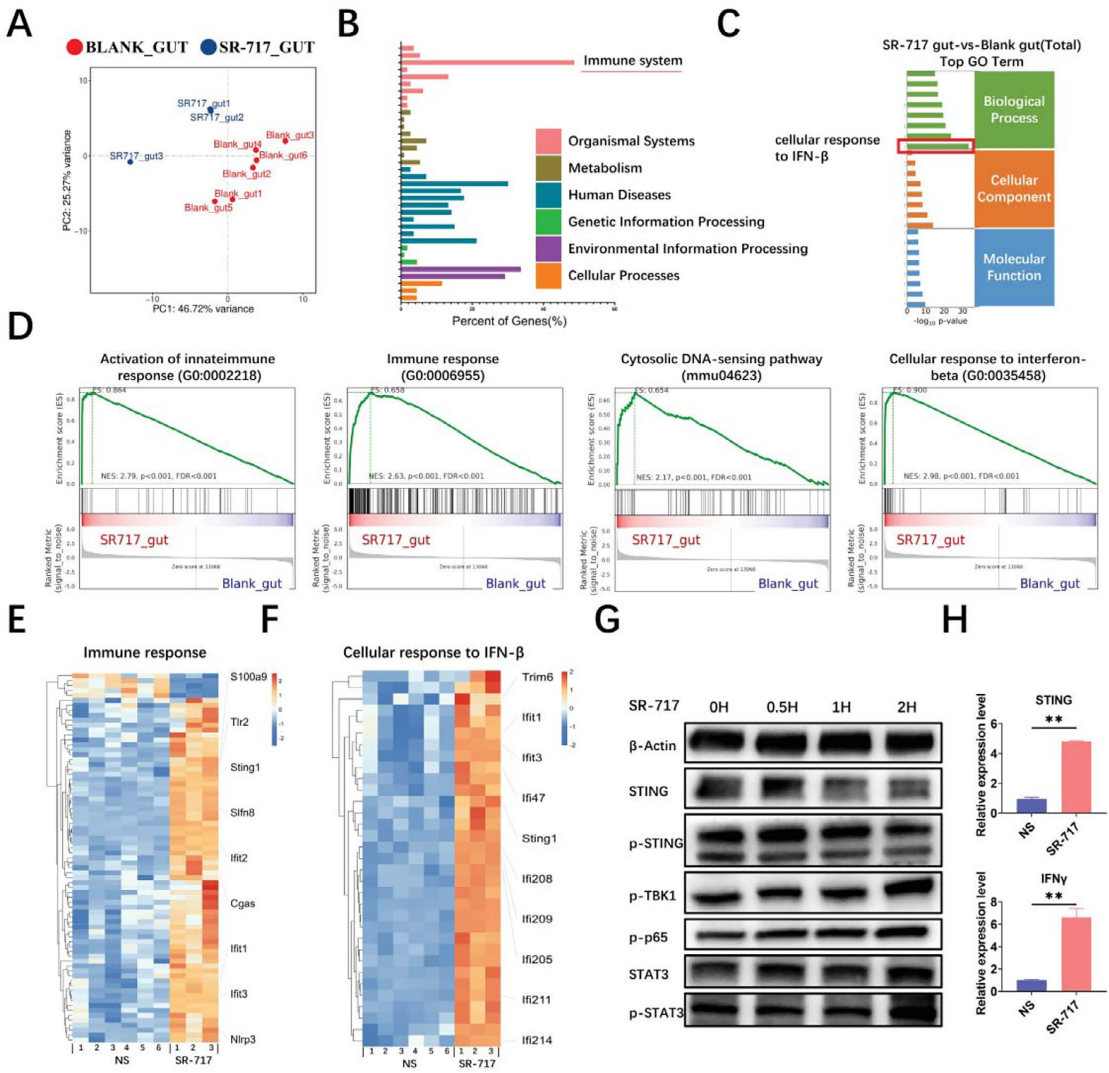
SR‐717 activates the immune system via STING‐IFN signaling. (A) PCA analysis in the intestines of mice treated in the presence or absence of SR‐717. (B) The KEGG Pathway Classification of mice treated in the presence or absence of SR‐717. (C) GO enrichment in the intestines of mice treated in the presence or absence of SR‐717. (D) GSEA in the intestines of mice treated in the presence or absence of SR‐717. (E) Heatmap of DEGs in immune response. (F) Heatmap of DEGs in cellular response to interferon‐beta. (G) Western blot analysis of activation of the cGAS‐STING signaling pathways in MODE‐K cells stimulated with SR‐717. (H) RNA obtained from mice intestinal tissues was used to perform the qRT‐PCR of STING and IFNγ(*n* = 3). Data are represented as mean ± SEM. **p < 0.01.

To explore the intestinal effects of SR‐717, RNA‐sequence analysis of mouse intestine was conducted in the presence or absence of SR‐717 (6:3). Principal‐coordinate analysis (PCoA) showed significant differences in intestinal genes between NS and SR‐717 mice (Figure 4A). We next performed GO analysis and KEGG analysis of the DEGs. In the KEGG analysis, we found that DEGs are primarily concentrated in the immune system (Figure 4B, Supporting Information S3). According to the GO analysis, DEGs are mainly enriched in the cellular response to interferon‐beta in the Biological Process (BP) enrichment terms (Figure 4C, Supporting Information S4). In addition, we performed Gene Set Enrichment Analysis (GSEA) on the whole genome, which showed that differentially expressed genes were mainly enriched in the activation of innate immune response (GO:0002218), immune response (GO:0006955), Cytosolic DNA‐sensing pathway(mmu04623) and cellular response to interferon‐beta (GO:0035458) pathways (Figure 4D, Supporting Information S5–S8). In addition, the DEGs in immune response (GO:0006955) and cellular response to interferon‐beta (GO:0035458) were shown as heatmaps (Figure 4E,F, Supporting Information S9 and S10). In addition, we found that SR‐717 significantly up‐regulated STING by extracting MODE‐K for Western blot analysis (Figure 4G). This result was also verified by extracting mice intestinal RNA for qRT‐PCR (Figure 4H). These results demonstrate that SR‐717 activates the immune system via STING‐IFN signaling.

### 3.5 The Radioprotective Effect of SR‐717 Was Dependent on IL‐6

**FIGURE 5:**
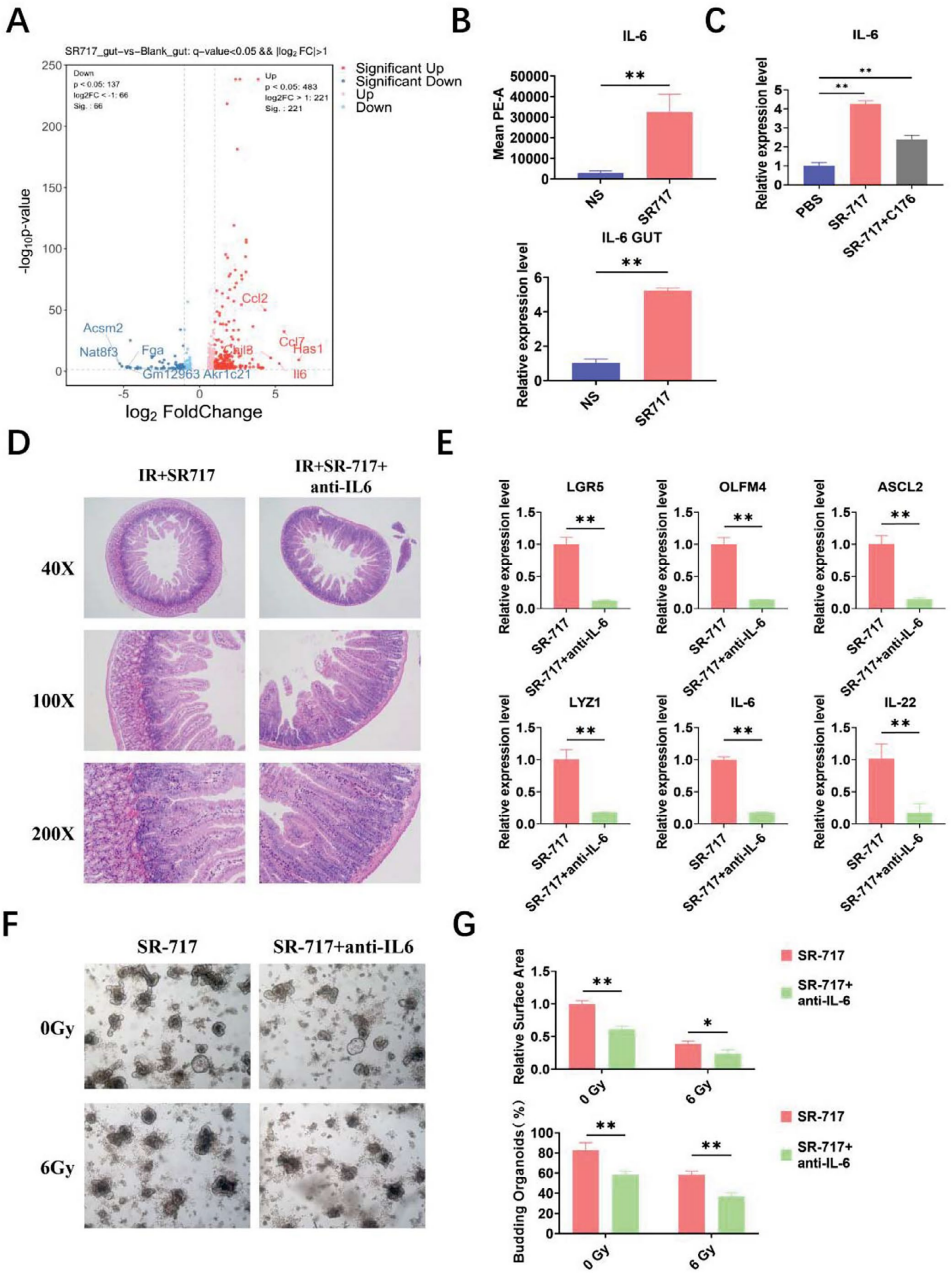
The intestinal radioprotection of SR‐717 depends on IL‐6. (A) Volcano plot analysis of gene expression of mice treated in the presence or absence of SR‐717 by RNA‐seq in mice intestines. (B) Serum levels of IL‐6 in mice, and mRNA levels in the mice intestine after IR exposure (*n* = 3). (C) mRNA levels of IL‐6 in the mice intestine after IR exposure(*n* = 3). (D) Pathological images of the intestine in mice 3.5 days after 9.5 Gy irradiation(*n* = 3). (E) mRNA levels including LGR5, OLFM4, ASCL2, LYZ1, IL‐6, IL‐22 in the mice intestine after IR exposure(*n* = 3). (F) Representative images of intestinal organoid growth after SR‐717 or SR‐717 + anti‐IL‐6 stimulation. (G) Relative area and budding rate of intestinal organoids. Data are represented as mean ± SEM. *p < 0.05, **p < 0.01.

By screening RNA‐Seq differential genes, we found that the expression of IL‐6, CCL2, and CCL7 was significantly increased in the intestine of mice after SR‐717 treatment (Figure 5A, Supporting Information S11). Next, the RNA‐Seq results were verified by flow cytometry and qRT‐PCR. We found that IL‐6 was up‐regulated in serum and intestine of mice (Figure 5B). Moreover, we used the STING signaling inhibitor C176 to assess the change of IL‐6. The qRT‐PCR result suggested that the expression of IL6 in the C176 group was significantly lower than that in the SR‐717 group, but significantly higher than the control group, suggesting that there may be other signaling pathways involved in the activation of IL‐6 (Figure 5C). Next, anti‐IL‐6 was used to determine whether SR‐717‐mediated IL‐6 induction was required for SR‐717 against IR‐induced intestinal damage. The results illustrated that anti‐IL‐6 significantly reduced the radioprotective effect of SR‐717. The villi damage of the intestines was aggravated after anti‐IL‐6 treatment (Figure 5D). The mRNA level of ISCs markers and ISCs regeneration related genes (LGR5, OLFM4, ASCL2, LYZ1, IL‐6, IL‐22) in the intestine was significantly decreased (Figure 5E). Moreover, intestinal organoids also showed the similar results (Figure 5F). The relative area and the percent of budding intestinal organoids were decreased significantly after treatment with IL‐6 inhibitor (Figure 5G). In sum, these results demonstrated that IL‐6 plays a central role in the regeneration of ISCs mediated by SR‐717, and SR‐717 may promote ISCs regeneration through up‐regulation of IL‐6.

### 3.6 TLR2 Signaling Pathway May Play a Critical Role in the Anti‐Radiation Activity of SR‐717

**FIGURE 6:**
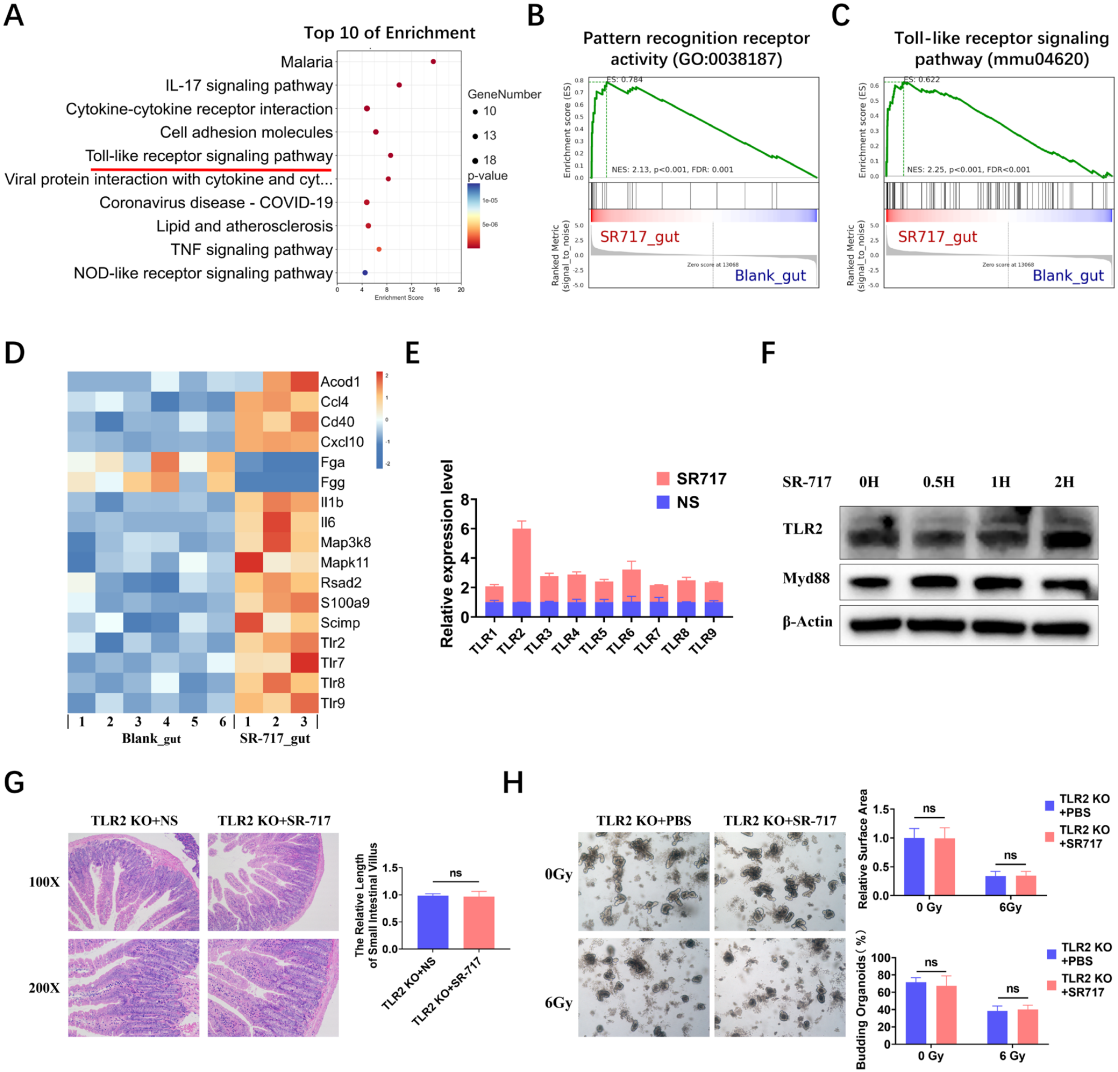
SR‐717 plays a role in intestinal radiation protection through TLR2 signaling pathway. (A) The KEGG pathway top 10 (Total) enrichment in the intestines of mice treated in the presence or absence of SR‐717. (B) GSEA of pattern recognition receptor activity. (C) GSEA of Tolls signaling pathway. (D) Heatmap of Toll‐like receptor signaling pathway genes. (E) RNA obtained from mice intestinal was used to perform the qRT‐PCR (*n* = 3). (F) Western blot analysis of activation of the TLR2 signaling pathways in MODE‐K cells stimulated with SR‐717 (*n* = 3). (G) TLR2 KO mice were treated with SR‐717 or saline before 9.5 Gy TBI. Pathological images and statistical analysis of the intestine in mice (*n* = 3). (H) Organoid extracted from TLR2 KO mice, were then exposed to SR‐717 or PBS. And the relative area and budding condition of intestinal organoids. Data are represented as mean ± SEM. ns, non‐significance.

With the aim of further exploring the mechanism of intestinal radioprotection by SR‐717, we further analyzed the RNA‐seq results. Interestingly, KEGG results suggested that the Toll signaling pathway was obviously enriched in the SR‐717 group (Figure 6A, Supporting Information S12). GSEA also showed that differentially expressed genes were mainly enriched in pattern recognition receptor activity (Figure 6B, Supporting Information S13) and the Toll signaling pathway (Figure 6C, Supporting Information S14). The heatmap of DEGs in the Toll signaling pathway revealed that TLR2, TLR7, TLR8, and TLR9 were increased after SR‐717 treatment (Figure 6D, Supporting Information S15). Moreover, qRT‐PCR results revealed that TLR2 was the most significantly upregulated gene after SR‐717 stimulation (Figure 6E). In addition, we found that SR‐717 significantly upregulated TLR2 and Myd88 (Figure 6F). In fact, a variety of cells in the intestinal epithelium could express TLRs. The activation of TLRs promoted the expression of downstream genes, which means that the activation of the TLR2/Myd88 signaling pathway was not limited to immune cells. Moreover, we examined the changes in immune cells in our work, such as dendritic cells (DCs). DCs were increased in the intestine after SR‐717 treatment (Figure S2), suggesting that DCs were involved in and contributed to the activation of TLR2/Myd88. Based on this, we used TLR2 KO mice to explore the role of TLR2 in the radioprotection of SR‐717. HE staining manifested that the radioprotection of SR‐717 was distinctly reduced in TLR2 KO mice, and there was a few differences in intestinal villi length between the SR‐717 group and the control group (Figure 6G). At the same time, we extracted the crypts of TLR2 KO mice for intestinal organoids culture and found that the relative area and budding rate of the SR‐717 group were similar to those of the control group (Figure 6H). These results indicated that the TLR2 signaling pathway may play a critical role in the anti‐radiation activity of SR‐717.

## 4 Discussion

Nuclear science and technology are widely used in the fields of medical treatment, nuclear energy, national defense, and military industry, and actively promote the development of the national economy. However, nuclear radiation can cause biological cell damage, destruction, and even cancer, which poses a great threat to human life safety [18, 19]. However, there is still a lack of effective methods to prevent and treat acute intestinal radiation sickness. As a result, it can never be overemphasized to explore the key mechanism of radiation damage and find new radioprotective agents.

Intestinal stem cells are the source of various intestinal epithelial cells. Intestinal stem cells, located in the base of intestinal crypts, replace damaged intestinal cells through proliferation and differentiation when intestinal cells are damaged or aged [20]. These newly generated cells move up the branched structure of the crypt and gradually differentiate into different types of intestinal cells, such as absorbent cells, secretory cells, and intestinal epithelial cells [21, 22]. The intestine is in direct contact with the external environment and is therefore susceptible to chemical or radiation‐induced damage [23, 24]. When the intestine is stimulated by a large dose of radiation, a large number of intestinal stem cells will die, resulting in irreparable damage to the intestine. Comprehending the regulatory mechanisms of ISCs is essential to preventing and treating IR‐induced intestinal injury.

STING, as a key signal transduction molecule of the innate immune response, plays an indispensable role in regulating the generation of spontaneous anti‐tumor immune response in vivo. By recognizing abnormal DNA and activating a series of signaling cascades, it not only promotes the production of type I interferons, but also plays a regulatory role in the function of immune cells such as dendritic cells and T cells.

The role of STING is particularly important in the intestinal environment [25], because the intestine is one of the organs most in contact with the external environment and has a rich microbial community. By sensing the DNA released by gut microbes, STING regulates the host's response to these microbes and helps maintain the balance of the gut microbiota [26, 27]. Activation of STING can boost the production of anti‐inflammatory cytokines that help prevent an excessive immune response, which is particularly important in intestinal diseases including inflammatory bowel disease (IBD) [26]. The signal of STING can enhance the tight connections of intestinal epithelial cells, thereby keeping the integrity of the intestinal barrier and preventing the invasion of pathogens and harmful substances. However, in some cases, overactivation of STING may lead to an increased inflammatory response, which is related to the occurrence and development of intestinal inflammatory diseases such as IBD [25]. STING signaling may also affect metabolic pathways in intestinal cells, including the ability of intestinal epithelial cells to secrete mucus, which is critical for protecting the gut from pathogens.

In this study, we found that STING agonist SR‐717 has a favorable radioprotective effect. SR‐717 can significantly increase the survival time of mice after irradiation, and to a certain extent, it can alleviate the hematopoietic injury caused by radiation, improve cell viability, and inhibit cell apoptosis. Moreover, intestinal pathology and immunofluorescence analysis showed that SR‐717 could alleviate the intestinal damage caused by radiation and up‐regulate the expression of intestinal stem cells, indicating that SR‐717 may play a radioprotective role by promoting the regeneration of intestinal stem cells after IR.

Intestinal organoids can simulate the structure and function of the intestine and can be used to study the self‐renewal, differentiation, and functional regulation of intestinal stem cells [16]. Through the intestinal organoid experiment, we found that SR‐717 significantly improved the production efficiency of intestinal organoids, with a larger surface area of intestinal organoids and a higher budding rate in the SR‐717 group. Moreover, by extracting intestinal organoid RNA for qRT‐PCR, we found that the mRNA level of intestinal stem cells in the SR‐717 group was significantly increased, proving that SR‐717 significantly promoted the ISCs regeneration in intestinal organoids after irradiation.

To further verify the mechanism of SR‐717 radiation protection, we performed RNA‐Seq technology to explore the mechanism of SR‐717 mediated intestinal radioprotection. The results suggest that SR‐717activates the immune system via STING‐IFNβ. Within the cell, STING is usually located on the endoplasmic reticulum (ER) and the Golgi membrane [28]. When cells detect invading pathogen DNA, STING migrates from the ER to the ER‐Golgi intermediate compartment, interacting with tank‐binding protein (TBK1) and transcription factor IRF3, resulting in phosphorylation and dimerization of IRF3. Activated IRF3 enters the nucleus and promotes the expression of type I interferon and other inflammation‐related genes [29]. The STING pathway plays a key role in the immune response, especially in anti‐tumor immunity and anti‐infection immunity. STING agonists recruit more NK cells and T cells, enhance the killing effect of immune cells on metastatic cancer cells, and inhibit metastatic outbreaks [30]. Meanwhile, STING not only participates in the immune response but also acts as a cell‐intrinsic metabolic checkpoint to limit aerobic glycolysis by targeting hexokinase 2 (HK2), thereby promoting anti‐tumor immunity in vivo [31]. Our results indicate that SR‐717 significantly upregulates the expression of STING and IFNγ in the mouse intestine, thereby activating immunity. Further analysis of the sequencing results showcased that IL‐6 was significantly upregulated by SR‐717. IL‐6 is indispensable for the damaged intestinal epithelial cells to survive, and the burst of IL‐6 expression was required to stimulate intestinal epithelial proliferation [32, 33, 34]. Studies show that blocking IL‐6 signaling exacerbates acute intestinal injury and late intestinal injury after focal irradiation [32]; Deficiency of IL‐6 in mice leads to increased activation of pro‐apoptotic and necrotic pathways in intestinal epithelial cells after injury [35]. Based on the results of RNA‐Seq and the above studies, we attempted to further explore the role of IL‐6 in SR‐717 against IR‐induced intestinal injury. We first assessed IL‐6 levels in the intestine and serum. In addition, intestinal radiological protection of SR‐717 was significantly inhibited after the use of IL‐6 inhibitors. These results suggest that intestinal radiological protection of SR‐717 depends on IL‐6.

Further analyzing the sequencing results, we found that SR‐717 significantly upregulated TLR2. For the past few years, some studies have reported that TLR is key in radiation protection [9, 36, 37]. TLR2 (Toll‐like receptor 2) is a pattern recognition receptor that is of vital importance in the human immune system and belongs to the Toll‐like receptor (TLRs) family [38]. TLRs are a key component of the innate immune system, and they are able to recognize conserved molecular patterns of microorganisms, such as lipopolysaccharides of bacteria, RNA of viruses, etc., to activate the immune response [39]. In our previous research, we found that TLR2 plays a key role in radiation resistance in vivo [40, 41]. TLR2 KO mice were more prone to radiation‐induced death when compared with WT mice [42].

Irradiated mice intestinal HE data showed that SR‐717 had little or no radioprotective effect on the intestines of TLR2 KO mice, and intestinal organoid technology analysis also showed that SR‐717 had no radiation protection effect on TLR2 KO mice. These results suggest that SR‐717 may play a radioprotective role in radiation‐induced intestinal injury by activating the TLR2 signaling pathway.

In conclusion, in this study, SR‐717 as a STING agonist can effectively improve the survival rate of IR‐treated mice and protect the intestine from IR‐induced damage. The RNA‐Seq results indicated that the radioprotection mechanism of SR‐717 was achieved through the TLR2 signaling pathway, and its intestinal radioprotection was dependent on IL‐6. In the present study, in addition to exploring the protective effects of SR‐717, we have in fact indirectly demonstrated the non‐target effects of small molecule agonists of STING, which not only suggests that SR‐717 can activate TLR2, but also suggests that targeted activation of STING can likewise up‐regulate IL‐6.

## Author Contributions

Jicong Du, Jianming Cai, and Fu Gao designed the study. Duo Fang, Wanli Duan, Liao Zhang, and Jianpeng Zhao performed the experiments. Xuanlu Zhai, Jiayan Fang, Keer Jiang, Yue Fu, Lan Fang, and Lu Pei were responsible for the collection of data and interpretation in the animal experiments. Cong Liu, Jicong Du, Jianming Cai, and Fu Gao analyzed the data. Jicong Du and Cong Liu supported fund assistance. All authors read and approved the final manuscript.

## Ethics Statement

All animal experiments conformed to the National Institute of Health Guide for the Care and Use of Laboratory Animals' (NIH Publication No. 85‐23, National Academy Press, Washington, DC, revised 1996), with the approval of the Laboratory Animal Center of the Naval Medical University, Shanghai.

## Consent

Written informed consent for publication was obtained from all participants.

## Conflicts of Interest

The authors declare no conflicts of interest.

## Supporting information


Data S1.

## Data Availability

All data generated or analyzed during this study were included in this published article and its Supporting Information S1.

## References

[1] A. M. Mowat and W. W. Agace , “Regional Specialization Within the Intestinal Immune System,” Nature Reviews. Immunology 14 (2014): 667–685, 10.1038/nri3738. 25234148

[2] J. Beumer and H. Clevers , “Cell Fate Specification and Differentiation in the Adult Mammalian Intestine,” Nature Reviews. Molecular Cell Biology 22 (2021): 39–53, 10.1038/s41580-020-0278-0. 32958874

[3] N. Barker , A. van Oudenaarden , and H. Clevers , “Identifying the Stem Cell of the Intestinal Crypt: Strategies and Pitfalls,” Cell Stem Cell 11 (2012): 452–460, 10.1016/j.stem.2012.09.009. 23040474

[4] C. Metcalfe , N. M. Kljavin , R. Ybarra , and F. J. de Sauvage , “Lgr5+ Stem Cells Are Indispensable for Radiation‐Induced Intestinal Regeneration,” Cell Stem Cell 14 (2014): 149–159, 10.1016/j.stem.2013.11.008. 24332836

[5] P. W. Tetteh , O. Basak , H. F. Farin , et al., “Replacement of Lost Lgr5‐Positive Stem Cells Through Plasticity of Their Enterocyte‐Lineage Daughters,” Cell Stem Cell 18 (2016): 203–213, 10.1016/j.stem.2016.01.001. 26831517

[6] M. Jiang , P. Chen , L. Wang , et al., “cGAS‐STING, an Important Pathway in Cancer Immunotherapy,” Journal of Hematology & Oncology 13 (2020): 81, 10.1186/s13045-020-00916-z. 32571374 PMC7310007

[7] A. Li , M. Yi , S. Qin , Y. Song , Q. Chu , and K. Wu , “Activating cGAS‐STING Pathway for the Optimal Effect of Cancer Immunotherapy,” Journal of Hematology & Oncology 12 (2019): 35, 10.1186/s13045-019-0721-x. 30935414 PMC6444510

[8] S. R. Paludan and A. G. Bowie , “Immune Sensing of DNA,” Immunity 38 (2013): 870–880, 10.1016/j.immuni.2013.05.004. 23706668 PMC3683625

[9] B. J. Leibowitz , G. Zhao , L. Wei , et al., “Interferon b Drives Intestinal Regeneration After Radiation,” Science Advances 7 (2021): eabi5253, 10.1126/sciadv.abi5253. 34613772 PMC8494436

[10] T. J. Hayman , M. Baro , T. MacNeil , et al., “STING Enhances Cell Death Through Regulation of Reactive Oxygen Species and DNA Damage,” Nature Communications 12 (2021): 2327, 10.1038/s41467-021-22572-8. PMC8055995 33875663

[11] S. Du , G. Chen , B. Yuan , et al., “DNA Sensing and Associated Type 1 Interferon Signaling Contributes to Progression of Radiation‐Induced Liver Injury,” Cellular & Molecular Immunology 18 (2021): 1718–1728, 10.1038/s41423-020-0395-x. 32203191 PMC8245603

[12] E. N. Chin , C. Yu , V. F. Vartabedian , et al., “Antitumor Activity of a Systemic STING‐Activating Non‐Nucleotide cGAMP Mimetic,” Science 369 (2020): 993–999, 10.1126/science.abb4255. 32820126

[13] T. Sato , J. H. van Es , H. J. Snippert , et al., “Paneth Cells Constitute the Niche for Lgr5 Stem Cells in Intestinal Crypts,” Nature 469 (2011): 415–418, 10.1038/nature09637. 21113151 PMC3547360

[14] L. G. van der Flier , A. Haegebarth , D. E. Stange , M. van de Wetering , and H. Clevers , “OLFM4 Is a Robust Marker for Stem Cells in Human Intestine and Marks a Subset of Colorectal Cancer Cells,” Gastroenterology 137 (2009): 15–17, 10.1053/j.gastro.2009.05.035. 19450592

[15] S. Yu , K. Tong , Y. Zhao , et al., “Paneth Cell Multipotency Induced by Notch Activation Following Injury,” Cell Stem Cell 23, no. 1 (2018): 46–59, 10.1016/j.stem.2018.05.002. 29887318 PMC6035085

[16] T. Takahashi , K. Fujishima , and M. Kengaku , “Modeling Intestinal Stem Cell Function With Organoids,” International Journal of Molecular Sciences 22 (2021): 10912, 10.3390/ijms222010912. 34681571 PMC8535974

[17] J. Sprangers , I. C. Zaalberg , and M. M. Maurice , “Organoid‐Based Modeling of Intestinal Development, Regeneration, and Repair,” Cell Death and Differentiation 28 (2021): 95–107, 10.1038/s41418-020-00665-z. 33208888 PMC7852609

[18] J. S. Parrish and G. Seda , “Disasters Resulting From Radiologic and Nuclear Events,” Critical Care Clinics 35 (2019): 619–631, 10.1016/j.ccc.2019.06.005. 31445609

[19] R. R. Babb , “Radiation Proctitis: A Review,” American Journal of Gastroenterology 91 (1996): 1309–1311. 8677984

[20] N. Barker , J. H. van Es , J. Kuipers , et al., “Identification of Stem Cells in Small Intestine and Colon by Marker Gene Lgr5,” Nature 449 (2007): 1003–1007, 10.1038/nature06196. 17934449

[21] J. Beumer and H. Clevers , “Regulation and Plasticity of Intestinal Stem Cells During Homeostasis and Regeneration,” Development 143 (2016): 3639–3649, 10.1242/dev.133132. 27802133

[22] N. Barker , M. van de Wetering , and H. Clevers , “The Intestinal Stem Cell,” Genes & Development 22 (2008): 1856–1864, 10.1101/gad.1674008. 18628392 PMC2735277

[23] Y. Li , J. Dong , H. Xiao , et al., “Gut Commensal Derived‐Valeric Acid Protects Against Radiation Injuries,” Gut Microbes 11 (2020): 789–806, 10.1080/19490976.2019.1709387. 31931652 PMC7524389

[24] J. Choi and L. H. Augenlicht , “Intestinal Stem Cells: Guardians of Homeostasis in Health and Aging Amid Environmental Challenges,” Experimental & Molecular Medicine 56 (2024): 495–500, 10.1038/s12276-024-01179-1. 38424189 PMC10985084

[25] J. Wang , N. Yao , Y. Chen , X. Li , and Z. Jiang , “Research Progress of cGAS‐STING Signaling Pathway in Intestinal Diseases,” International Immunopharmacology 135 (2024): 112271, 10.1016/j.intimp.2024.112271. 38762923

[26] M. C. C. Canesso , L. Lemos , T. C. Neves , et al., “The Cytosolic Sensor STING Is Required for Intestinal Homeostasis and Control of Inflammation,” Mucosal Immunology 11, no. 3 (2018): 820–834, 10.1038/mi.2017.88. 29346345

[27] R. Zhang , C. Yu , H. J. Zeh , et al., “Nuclear Localization of STING1 Competes With Canonical Signaling to Activate AHR for Commensal and Intestinal Homeostasis,” Immunity 56 (2023): 2736–2754, 10.1016/j.immuni.2023.11.001. 38016467 PMC10842782

[28] H. Ishikawa and G. N. Barber , “STING Is an Endoplasmic Reticulum Adaptor That Facilitates Innate Immune Signalling,” Nature 455 (2008): 674–678, 10.1038/nature07317. 18724357 PMC2804933

[29] C. Chen and P. Xu , “Cellular Functions of cGAS‐STING Signaling,” Trends in Cell Biology 33 (2023): 630–648, 10.1016/j.tcb.2022.11.001. 36437149

[30] J. Hu , F. J. Sánchez‐Rivera , Z. Wang , et al., “STING Inhibits the Reactivation of Dormant Metastasis in Lung Adenocarcinoma,” Nature 616 (2023): 806–813, 10.1038/s41586-023-05880-5. 36991128 PMC10569211

[31] L. Zhang , C. Jiang , Y. Zhong , et al., “STING Is a Cell‐Intrinsic Metabolic Checkpoint Restricting Aerobic Glycolysis by Targeting HK2,” Nature Cell Biology 25 (2023): 1208–1222, 10.1038/s41556-023-01185-x. 37443289 PMC11232535

[32] B. I. Bell , S. Koduri , C. Salas Salinas , et al., “Interleukin 6 Signaling Blockade Exacerbates Acute and Late Injury From Focal Intestinal Irradiation,” International Journal of Radiation Oncology, Biology, Physics 103 (2019): 719–727, 10.1016/j.ijrobp.2018.10.007. 30336264 PMC7699458

[33] K. A. Kuhn , N. A. Manieri , T. C. Liu , and T. S. Stappenbeck , “IL‐6 Stimulates Intestinal Epithelial Proliferation and Repair After Injury,” PLoS One 9 (2014): e114195, 10.1371/journal.pone.0114195. 25478789 PMC4257684

[34] S. Grivennikov , E. Karin , J. Terzic , et al., “IL‐6 and Stat3 Are Required for Survival of Intestinal Epithelial Cells and Development of Colitis‐Associated Cancer,” Cancer Cell 15 (2009): 103–113, 10.1016/j.ccr.2009.01.001. 19185845 PMC2667107

[35] X. Jin , T. A. Zimmers , Z. Zhang , R. H. Pierce , and L. G. Koniaris , “Interleukin‐6 Is an Important In Vivo Inhibitor of Intestinal Epithelial Cell Death in Mice,” Gut 59 (2010): 186–196, 10.1136/gut.2008.151175. 19074180

[36] S. Kumar and R. Kumar , “Role of Acemannan O‐Acetyl Group in Murine Radioprotection,” Carbohydrate Polymers 207 (2019): 460–470, 10.1016/j.carbpol.2018.12.003. 30600029

[37] S. Sanguri and D. Gupta , “Mannan Oligosaccharide Requires Functional ETC and TLR for Biological Radiation Protection to Normal Cells,” BMC Cell Biology 19 (2018): 9, 10.1186/s12860-018-0161-4. 29945545 PMC6020349

[38] S. Akira , S. Uematsu , and O. Takeuchi , “Pathogen Recognition and Innate Immunity,” Cell 124 (2006): 783–801, 10.1016/j.cell.2006.02.015. 16497588

[39] T. Kawai and S. Akira , “The Role of Pattern‐Recognition Receptors in Innate Immunity: Update on Toll‐Like Receptors,” Nature Immunology 11 (2010): 373–384, 10.1038/ni.1863. 20404851

[40] J. Du , L. Fang , J. Zhao , et al., “Zymosan‐A Promotes the Regeneration of Intestinal Stem Cells by Upregulating ASCL2,” Cell Death & Disease 13 (2022): 884, 10.1038/s41419-022-05301-x. 36266266 PMC9585075

[41] L. Fang , Y. Cheng , D. Fang , et al., “CL429 Enhances the Renewal of Intestinal Stem Cells by Upregulating TLR2‐YAP1,” International Immunopharmacology 138 (2024): 112614, 10.1016/j.intimp.2024.112614. 38972212

[42] F. Gao , C. Zhang , C. Zhou , et al., “A Critical Role of Toll‐Like Receptor 2 (TLR2) and Its' In Vivo Ligands in Radio‐Resistance,” Scientific Reports 5 (2015): 13004, 10.1038/srep13004. 26268450 PMC4534783

