# Supplementary figures and images for: SR‐717, a Non‐Nucleotide STING Agonist, Displayed Anti‐Radiation Activity in a IL‐6 Dependent Manner
Source: FASEB J. 2025 May 31;39(11):e70644. doi: 10.1096/fj.202403127R (PMC12125615; doi:10.1096/fj.202403127R)

FIGURE 4G

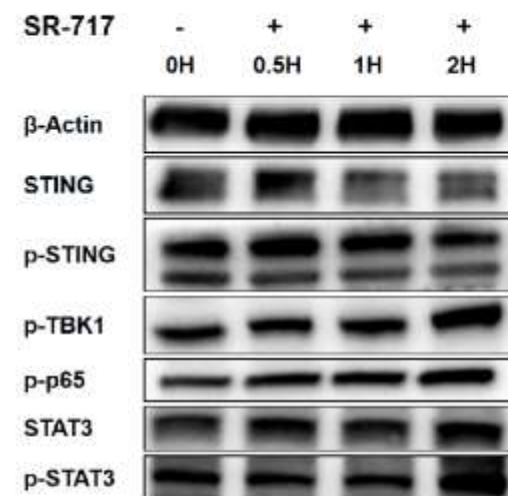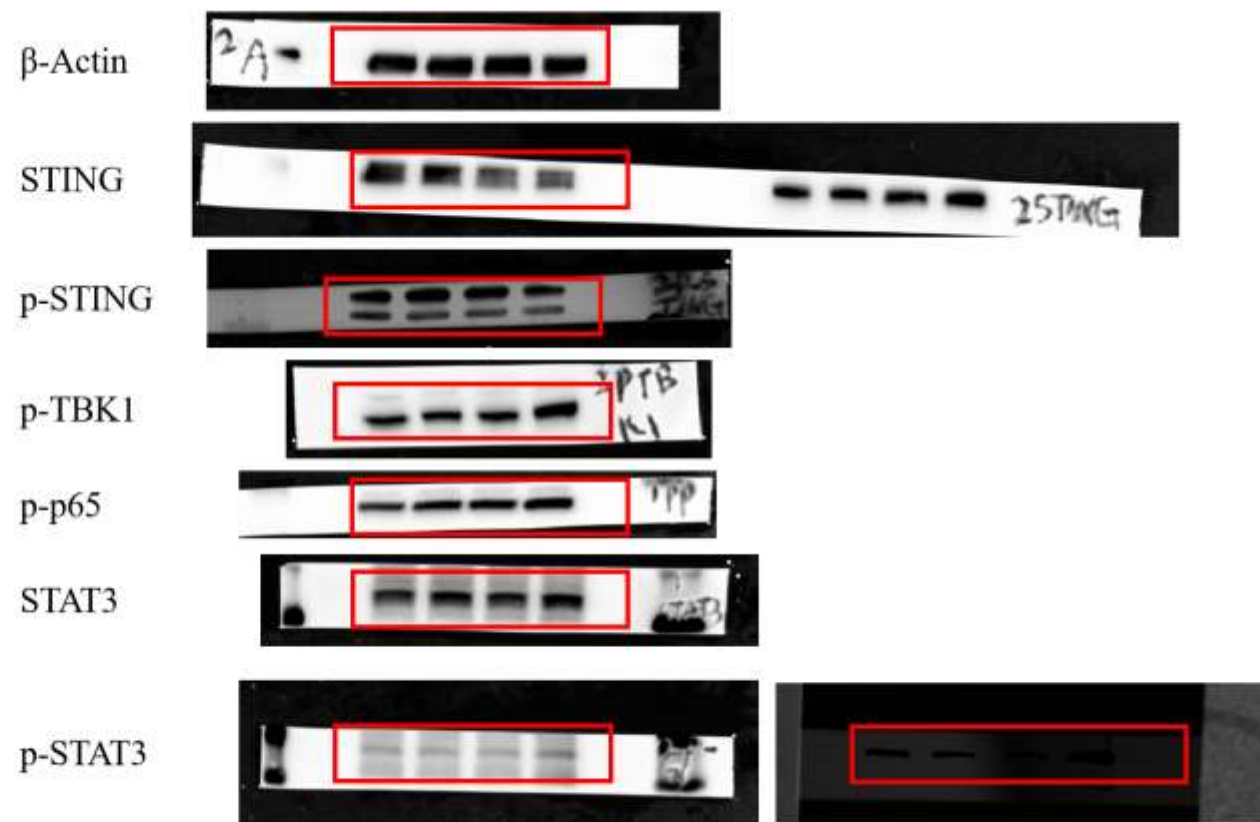

FIGURE 6F

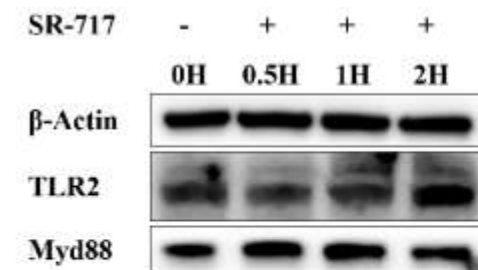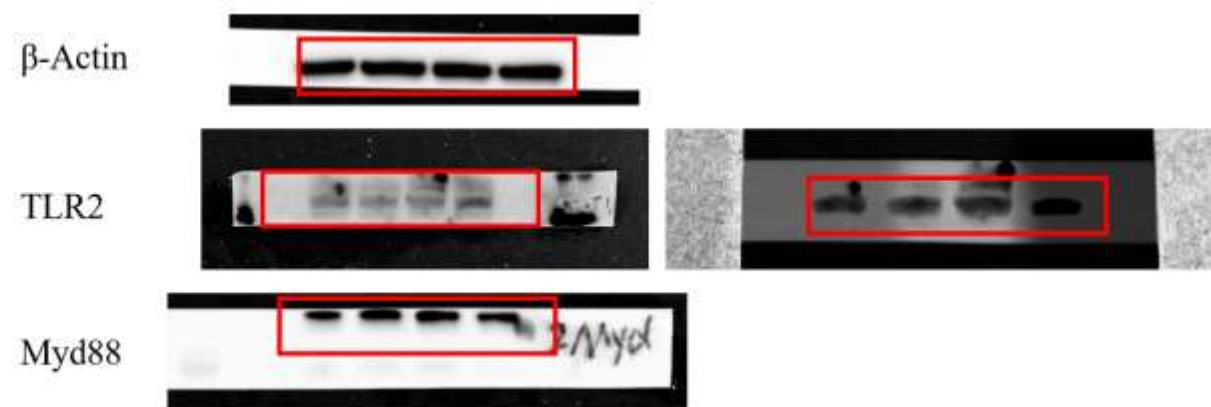

Supplement: Supplementary file 1 — Data S1. [file FSB2-39-e70644-s001.zip › fsb270644-sup-0001-FigureS1.pdf]
